# The Internet Knows More Than My Physician: Qualitative Interview Study of People With Rare Diseases and How They Use Online Support Groups

**DOI:** 10.2196/39172

**Published:** 2022-08-25

**Authors:** Sadaf Ashtari, Adam Daniel Taylor

**Affiliations:** 1 California State University, Sacramento Sacramento, CA United States

**Keywords:** online peer support group, genetic disorders, pain management, Ehlers-Danlos syndrome, EDS, chronic pain, health care provider, pain mitigation techniques

## Abstract

**Background:**

Patients struggling with rare diseases may face challenges caused by care providers being unfamiliar with their condition. The life span of people with rare diseases may be the same as that of healthy people, but their quality of life is different. Patients with chronic pain are constantly looking for ways to mitigate their pain. Pain killers are not a permanent solution. In addition to the medical and nonmedical costs of rare diseases for both patients and health care providers, there is a need for sustainable sources of information that are available to help with pain and improve their quality of life, with the goal of reducing physician visits and hospital admissions.

**Objective:**

This study investigated the challenges that patients with genetic disorders face in managing their health conditions and finding disease-related information as well as the effect of online peer support groups on pain mitigation and care management.

**Methods:**

Interviews were conducted via Zoom between July 2021 and December 2021. Eligible participants were those who were aged >18 years, had a medical diagnosis of any type of Ehlers-Danlos syndrome (EDS) with chronic pain, and were members of any support group. Participants were recruited through an announcement in the research and survey section of The Ehlers-Danlos Syndrome Society web page. Interviews were analyzed using the framework approach. Data were systematically searched to identify patterns, analyze them, and identify themes. Interview audio files were transcribed and independently coded by two researchers (SA and AT). Through an iterative process, a final coding table was agreed upon by the researchers and used to thematically analyze the data.

**Results:**

We interviewed 30 participants (mean age 37.7, SD 15 years; n=28, 93% were women; n=23, 77% were residing in the United States). Thematic analysis revealed that participants (patients with EDS) were constantly in pain and most of them have not received accurate and timely diagnoses for many years. They expressed their challenges with health care providers regarding diagnosis and treatment, and complained about their providers’ lack of support and knowledge. Participants’ main sources of information were web-based searches, academic journals, The Ehlers-Danlos Syndrome Society web page, and online peer support groups on Facebook, Reddit, Twitter, and Instagram. Although pain killers, cannabis, and opioids are providing some pain relief, most patients (28/30, 93%) focused on nonmedical approaches, such as hot or ice packs, physical therapy, exercises, massage, mindfulness, and meditation.

**Conclusions:**

This study highlights the information gap between health care providers and patients with genetic disorders. Patients with EDS seek access to information from different web-based sources. To meet the needs of patients with genetic disorders, future interventions via web-based resources for improving the quality of care must be considered by health care professionals and government agencies.

## Introduction

### Background

More than 400 million people (30 million Americans) are living with one or more of the 7000 identified rare diseases. Rare diseases encompass many different disorders and symptoms, but approximately 72% of rare diseases are genetic [1]. In this study, our focus was on Ehlers-Danlos syndrome (EDS), a genetic disorder. It is an inherited connective tissue disorder that causes problems in the skin, blood vessels, bones, and other organs. Patients with EDS have to cope with complications such as joint hypermobility or dislocations and damaged skin for their entire life [2]. Similar to other rare diseases, a lack of scientific knowledge and information regarding EDS causes delays in diagnosis. On average, it takes 6 to 8 years for patients to obtain an accurate diagnosis of their disease. There are no Food and Drug Administration–approved drug treatments for >95% of all rare diseases [3]. Therefore, patients with genetic disorders are under considerable pressure to learn about their disease and how they can manage their pain [4]. On the basis of the recent survey results, compared with other diseases such as cancer, patients with genetic disorders did not feel sufficiently supported with issues related to mental health, navigating the health system, physical and daily living, patient care, and sexual needs [5]. The life span of people who are diagnosed with genetic disorders is often the same as that of healthy people, but the quality of their life is usually very different. There are no cures for genetic disorders; therefore, patients cope by mitigating pain and maximizing their quality of life [6].

The availability of health information on the web has increased dramatically over the past decade. A study in 2018 showed that adults in the United States looked on the web for health information 59% more than in 2013 [7]. The study also revealed that, currently, 55% of health care information seekers are relying more on the internet and web-based resources for their health-related information than 5 years ago. More than 67% of American health care information seekers mentioned that they look for health information on social media. The importance of online support groups has been studied among different people [8] with different diseases such as Parkinson disease [9], psychiatric disabilities [10], amyotrophic lateral sclerosis [11], breast cancer [12], chronic diseases [13,14], neuromuscular disorders [15], and alcoholism [16]. Several studies [12,13,17] have discussed the positive impact of online support groups and how patients can benefit from the emotional and community support obtained from such groups. Online support groups are also used for information support. People who faced similar problems can share possible solutions, suggest how to cope with symptoms, and provide information about their disease to other members of the group [11,18].

### Objective

This study investigated the approaches followed by patients with EDS to gain information about their disease, find solutions and treatments, and discover pain mitigation techniques. In addition, the effects of online peer support groups on pain mitigation and care management were examined. Information about the challenges faced by patients with genetic disorders with their health care providers is crucial for governments, researchers, health care regulations, and policy makers to be able to improve health care and population health management.

## Methods

### Overview

This study comprised qualitative, individual, semistructured interviews with patients with EDS to gather perspectives on the care management. Interviews were conducted to investigate the effectiveness of online peer support groups in managing the health conditions of patients with genetic disorders. Transcripts were analyzed using thematic analysis approach, which allows for both inductive and deductive themes to be explored using an iterative, constant comparative coding process.

### Ethics Approval

All research activities were approved by the institutional review board of the Sacramento State University (protocol number IRB-19-20-173).

### Participants

A total of 30 participants were recruited by advertising the study on The Ehlers-Danlos Syndrome Society web site. Eligible participants were those who were aged >18 years, had a medical diagnosis or a suspected case of any type of EDS with chronic pain, and were members of an online support group.

### Recruitment

The recruitment flyer and announcement letter were posted on The Ehlers-Danlos Syndrome Society website. Volunteers for the study were instructed to email the primary investigator to express their interest and learn more about the study. In response to those volunteers, the primary investigator (SA) of the study emailed the e-consent form, study details, time window for the appointment (in PST), and a request for their availability for a 30-minute interview. From July 2021 to December 2021, we received 57 emails and eventually scheduled appointments with 30 (53%) of them. The remaining volunteers (27/57, 47%) did not respond regarding their availability. The interviews were conducted via Zoom, with the participants’ permission to record the session.

### Data Collection

The semi-instructed interviews were conducted by the first author of the study (SA), using the question guide presented in Multimedia Appendix 1. The discussion began by sharing the experiences of the researcher (SA) as a patient with genetic disorder along with chronic pain and further discussions about the origins of this study. Participants could ask questions at any point during the interview. After explaining the background of the study, participants were asked several demographic questions, leading to questions about their experience with pain and EDS. Participants themselves tended to guide the conversation, and they explained in detail about their journey toward learning more about EDS. The interviewer provided prompts to pursue more detailed discussions on selected topics and help keep the discussions on track.

The main focus of the discussions, along with the research questions, was on (1) pain management techniques, (2) different sources of information about patient’s health, (3) use of online support groups in managing one’s health condition, and (4) health care providers’ roles. The average length of each interview was 45 minutes.

### Data Analysis

The interviews were audio-recorded and transcribed verbatim. The research team used inductive thematic approach to develop themes from the interview transcripts. In total, two authors (SA and AT) independently read the 30 transcripts several times to familiarize themselves with the data and identify themes. The 2 authors met to discuss their findings to resolve differences. After this analysis, the authors developed a thematic framework. They also coded all the transcripts using this thematic framework independently. The main topics from the interview guide were used to create the initial deductive codes. Deductive and inductive codes were identified and used, respectively, in our analysis. Then, the authors discussed the results to reach consensus and refine the main themes. NVivo (version 12.2.0; QSR International) was used in this study to organize the transcripts and facilitate analysis.

## Results

### Overview

Following our template analysis, seven main themes were identified based on patients’ experiences of living with genetic disorders and participating in online support groups and their efforts to find solutions for their pain and health conditions. Our themes include (1) patients with EDS disorders are constantly in pain; (2) challenges with health care providers regarding diagnosis and treatment; (3) lack of health care provider support; (4) searching for different sources of information; (5) collection of different pain management techniques; (6) finding disease treatments, lifestyle solutions, and shared experiences in online peer support groups; and (7) changes in health management as a result of participating in online support groups.

### Participant Characteristics

A total of 30 English-speaking participants were interviewed in 2021. Participant characteristics are reported in Table 1. Participants had a mean age of 37.7 years. Most participants (28/30, 93%) identified as women, with most of them residing in the United States (23/30, 77%). Our demographics reflect that of the general population with EDS, which is 73.9% women and 26.1% men [19,20]. Most participants (28/30, 93%) were White. In total, >65% of the participants had some college and graduate degree; however, <45% of them were currently working. Owing to chronic pain and health conditions, most of them (17/30, 57%) were temporarily disabled or can no longer work.

**Table 1:** Participants’ characteristics (N=30).

Characteristics			Values, n (%)
**Age (years)**			
	10-21	2 (7)	
	20-29	4 (13)	
	30-39	13 (43)	
	40-49	4 (13)	
	50-59	4 (13)	
	60-69	3 (10)	
**Gender**			
	Women	28 (93)	
	Nonbinary	2 (7)	
**Ethnicity and race**			
	African American or Black	1 (3)	
	Pacific Islander	1 (3)	
	White	28 (93)	
**Highest level of education**			
	High school	10 (33)	
	Bachelor degree	7 (23)	
	Master degree	10 (33)	
	PhD	3 (10)	
**Currently employed**			
	No	17 (57)	
	Yes	13 (43)	
**Country of residence**			
	International	7 (23)	
	United States	23 (77)	

### Patients With EDS Disorders Are Constantly in Pain

Patients with EDS are constantly in pain. They expressed their pain frequency as every day and all the time. The level of pain and its location varied among patients. All quotes are presented verbatim, but to improve readability, some filler words such as *like* and *um* were removed. In addition, the information in the bracket at the end of each quote refers to the interviewee’s information that we deidentified and coded. A participant described her pain as follows:

Every day, I would say that my normal pain level is probably a three to four that’s like my normal, but then whenever I’m having a really bad flare it could be up to like seven sometimes eight.

H-CA

The participants also showed their frustration of being in constant pain and their desperation to find a cure or treatment:

I am in pain everyday all the time, it’s pretty ridiculous.

E-CA

I am in pain every minute, every second, every hour, every day, non stop.

SH-U

I probably suffer about 20 to 30 sublocations and dislocations a day, I mean, I’ve always been in pain.

El-U

### Challenges With Health Care Providers Regarding Diagnosis and Treatment

Participants shared their difficulties in receiving timely and precise diagnosis from their health care providers. Most of them (25/30, 83%) declared that their disease remained as a mystery for many years before they were diagnosed with EDS:

I’ve always known there’s something different about me, but I was not formally diagnosed until February of this year. I’ve been regressing pretty steadily since March of 2019, but I did not have a formal diagnosis and so. I go to a lot of different doctors to try different things.

AM-CO

I live in a really rural area as well, so our medical care is pretty limited. You know I don’t think there’s any doctors here that really too familiar.

SA-TE

I’m so used to managing it on my own. Since I was diagnosed over 100 different doctors specialists in different areas. Doctors are completely useless, most of the time when I start listing my medical conditions I’ll get to the third or fourth one and their eyes glaze over and it’s like they just assume.

SA2-CA

Several participants (8/30, 27%) were misdiagnosed and had to visit different providers to understand their actual problem:

I’ve been hyper mobile since I was a young child and it got misdiagnosed as various things along the way, until we finally landed on what we now know.

PA-MI

The diagnosis was only recent probably within the last six months, but up until then everyone thought it was fibromyalgia, but I had been tested for Parkinson’s and for multiple sclerosis and for arthritis and every other kind of immunological disease that they could think of.

CH-NA

It’s actually more recent diagnosis. I was mistaken as with lupus for a long time.

EL-U

Given that participants had to deal with chronic pain for most of their life, they were eager to learn about their disease and treatment solutions through a trusted source such as a health care provider. Lack of knowledge and awareness among physicians and family members has made the life of patients with EDS more difficult:

My biggest pain problem was like my legs, and I remember at being two years old crying on the floor of a gas station and my mom’s like come on get up. You know because she didn’t believe me, and throughout my whole life I’ve had these leg pains that would come and go, and just nobody knew what it was you know, and I was 46 years old, when I first got diagnosed.

SA-SC

I have known about it all my conscious life. I have been working on trying to figure out what this hypermobility means and trying to get doctors and medical professionals help me understand what it means, and it’s been a long journey. I did the research myself and looked up medical articles. I took that to my doctor, and I said, “I think I have this” and she read it, looked at it and said, “you know, you are probably right.” So, I did it myself, that was in probably 2006, 2007.

KA-CA

### Lack of Health Care Provider Support

One of the main complaints from participants was the lack of support from their health care providers. Many participants (26/30, 87%) mentioned that their medical team was not supportive and did not trust them when they said they were in pain:

I’ve had issues and it’s kind of like, you may have this but it doesn’t really matter anyway because it’s having a lot of other issues that they couldn’t put together, so I’ve known about it for a long time but been diagnosed for about a year. Because that’s one of the biggest things right it’s like you have doctors that just don’t believe you or they don’t believe in EDS. And they’ll tell you it’s all in your head.

JU-KE

Just that fear of you know, having a doctor be like Oh, this is all in your head you’re just looking for attention you’re just a girl Blah Blah Blah like everything that I heard from like age seven. My friend said to do stuff to get doctors to take you seriously.

JE-BO

Several participants (10/30, 33%) expressed helplessness in their current situation and desire to find a way to manage their health in a less stressful manner:

I distinctly remember being kicked out of Tuft’s Ehlers-Danlos because I had been fainting left and right and they’re like you should try acupuncture or yoga and we think that this is a psychiatric problem.

JE-BO

I am scared that it is never going to heal right, and I will never have the strength that I used to have.

ER-U

### Searching for Different Sources of Information

Participants reported searching different sources for disease information and pain mitigation techniques. Approximately all participants (29/30, 97%) were actively searching the internet to learn about their problem and find solutions to manage their health. Among these resources, online peer support groups hosted by Facebook, Instagram, Twitter, Reddit, and Inspire were the most popular. Each of these platforms has its own tools and methods for distributing information:

I find the Facebook group very helpful because they are like people chain, for example one kind of antibiotics that we should not use they shared a name, so I know right now. I’m in local EDS group in Italy.

DA-IT

The nice thing about doing the Instagram awareness thing was a bunch of people were in my group message but I’m already friendly with them and they are like oh hey I have this too, and let’s chat and you know so I’ll go to them now and be hey have you tried blah blah blah.

JE-BO

It’s been helpful to be able to talk to other people that have it, too, because they go through what you go through, as opposed to like a medical professional that doesn’t go through it and they’re trying to help you arguing.

HA-CA

The subreddit for Ehlers-Danlos and be like hey does anybody know of a thing that’s useful for like this type of pain Has anybody had any luck with X or Y or Z.

JE-BO

Most participants (25/30, 83%) described their approaches to finding information about their disease. As a first step, they searched symptoms and problems on Google. Most of the time, Google directed them to scientific and scholarly pages from medical journals:

I go straight to the internet because that’s what my doctors are doing, you know even my doctors don’t know what they’re up against so there’s really no better.

CH-NA

I’ll just Google what the issue is and research that and then I usually bring that to my doctor, and I look more than often and look on PubMed for actual research.

AM-CO

Another trusted source of information is The Ehlers-Danlos Syndrome Society website. Participants rely heavily on the disease information found in that website:

I also usually go to the Ehlers-Danlos society. They have a lot of information and videos and stuff about EDS so I can usually find stuff there. The Ehlers-Danlos society has an LGBT support group and I go to that one exclusively because it’s so much better.

MI-SE

The Ehlers-Danlos society that is a really good place to get information.

KA-U

### Collection of Different Pain Management Techniques

Participants discussed the nonmedical pain management techniques that they learned through online support groups, web searches, and reading other patients’ experiences. Many nonmedical pain mitigation techniques that they use include hot and cold packs, mental therapy and meditation, topical creams, diet, braces, distraction, and salt baths:

Ice and heat are my best friend.

PA-MI

My greatest tool for dealing with the pain is mindfulness.

ME-DC

I also have some over the counter muscle rub that’s a help, I take a muscle relaxer every day because a lot of the pain that I have is from my muscles being so tense because they’re doing the job of my joints.

LY-NA

One thing that I am trying to do is take a look at my own nutrition and figure this out. You know, what is helpful, what should I avoid, and it’s been a long journey, but I think I am getting closer. Certain foods are irritating, some fruits make me inflamed and retain water and gain weight. The blood type diet is very helpful.

KA-CA

Participants shared their experiences with physical therapy, exercises, dry needling, and acupuncture for reducing their pain. They had both positive and negative experiences with physical therapy. They mentioned that the knowledge of the physical therapist and their familiarity with EDS are necessary for achieving a good result in pain reduction:

I’m going to physical therapy for the EDS and that’s been helping me a ton in terms of the pain. She started me in the pool which was super helpful.

JE-BO

When I first got diagnosed my doctor sent me for physical therapy. After about a month they said you’re not getting any better, so we can’t help you so that ended, because at the time they did not know how they can help me.

SA-SO

I get dry needling done. It makes such a difference to my functionality and my pain. It’s definitely worth finding the right person to do it.

SA-AU

Several participants (4/30, 13%) talked about pain management classes that they attended through their health care provider, but the result was not very satisfactory:

I was going to the pain management program. They do a lot of stuff like learning how to meditate and calm your system down. They don’t use any kind of painkillers. It’s all like the mind and body. The only issue with the pain management program is they’re not very educated on people with EDS, and so they were having me do everything that everybody else was doing and that could be very harmful to those of us with EDS.

HE-CA

Many participants (28/30, 93%), through trial and error and web-based research, found body positions that gave them less pain and great relief. They tried taping, stretching, moving, sitting, and resting in different positions:

I move myself every day. To start the circulation in the blood system, because then I don’t get stuck. Sleeping well it’s good.

LO-SW

It’s very important that I listened to my body and lay down when it says lay down.

LY-NA

What position can I put my body in to quiet it and so slowly but surely, I found things, to do the trick. When I got into where I had to keep moving around...

SU-NY

Approximately half of the participants (16/30, 53%) mentioned that they had used different drugs such as opioids and cannabis. However, many of them (10/30, 33%) expressed their concern about addiction or developed drug tolerance and could not receive the same relief in the long term:

The topical medical marijuana stuff with cannabidiol in it and essential oils, some of those are amazing.

SA-CA2

I don’t take any pain medication I had a very, very bad experience over multiple years of being on progressive levels of opioids.

SA-CA

I was taking opioids when I first got diagnosed. I took those for about three years, and I got off of them two reasons, first, I could tell my body was becoming too used to them, and you know I had to take more and more, but also it actually made my pain worse because you know my body got used to it.

SA-SO

Although participants named 23 different nonmedical pain mitigation techniques, pain killers were still on everyone’s list. For most of them (20/30, 67%), pain killers and anti-inflammatory medication were not sufficient. They have to use multiple techniques to reduce their pain and frustration. Several pain killers were being used: naltrexone, celecoxib, ibuprofen, meloxicam, carprofen, tylenol, and amitriptyline. They also mentioned using antidepressant medicines such as fluoxetine and diazepam or similar products for sleeping problems caused by their persistent pain.

### Finding Pain Solutions, Lifestyle Tips and Tricks, and Community Support Through Online Peer Support Groups

Most interviewees (24/30, 80%) were participating in online peer support groups to find pain management solutions, physician and product referrals, natural remedies, or tips and tricks that can make their life easier. They also benefited from sharing their experiences and hearing other patients’ stories:

I like when people have recommendations or like what works for them. Different kind of tips and tricks, I guess.

JE-AL

Information about my disorder like you dislocate your shoulder What do you do when your shoulder dislocates do you wear a brace like this do you tape it, how did you tape it yourself, do you use YouTube videos learning it like What do you do you know I mean.

MI-SE

I usually go on them like the Amazon recommendations I find those really helpful, especially when I’m trying to buy furniture. I have asked in the past about assistive devices, so you know it’s getting hard for me to turn door handles, anybody got a suggestion.

SA-AU

Several participants (7/30, 23%) mentioned their gratitude for being able to help and support other people:

I am able to help people because I see these people that are just starting on their journey or they are much younger than me and they’ve been diagnosed, so I can give them these tips that are all my best practices that you know.

SA-CA

Another reason why patients are participating in online peer support groups is the discovery of an empathetic community of people with the same health issues. They stated that being part of the EDS community improves their mental health:

It’s been helpful to be able to talk to other people that have it, too, because they go through what you go through, as opposed to like a medical professional that doesn’t go through it and they’re trying to help you.

HE-CA

I was like I’m normal within my subset of people, I’m the majority here, so that was kind of cracking me up because you’ve been the outcast or that you were the outlier, I guess your whole life.

SA-CA

### Changes in Health Management as a Result of Participating in Online Support Groups

Approximately all participants (29/30, 97%) reported that their health care management changed as a result of participating in online peer support groups. They agreed that they learned more about their condition and how to handle it after using online support groups. Being part of a group made them feel less alone, reduced their stress, and eventually helped with their pain reduction:

You know they’re a blessing and a curse I think they’re invasive at times and they’re a little demanding but they’re also incredibly helpful I don’t think I would have gotten as far along on my own journey with figuring out what was going on with me, it’s definitely helped me to take better care of myself really.

CH-NA

It makes me feel less alone, especially you know, being in you understand, you’re in your 30s and you’re dealing with all this chronic pain. These groups have also made me feel valid.

HE-CA

They help me remember that I am not a normal person. I cannot do some of the things that other people do, and I do have limits and those are good reminders of that. That oh yeah, I do have limits and oh yeah I shouldn’t feel bad that I could not eat pizza with all that cheese and gluten and you know, there is a reason why I am choosing to eat the way I eat, and exercise the way I exercise.

KA-CA

They described that their participation in online peer support groups helped them to be more vocal about their needs and start taking better care of themselves:

If I didn’t have the support group I wouldn’t be managing my health, the way that I am.

JU-KE

I’m a lot more proactive I know a lot more. I learned so much.

LO-UK

I’ve learned to be much more advocate for myself because before you know I found out about it, that I had a rare disorder, it was just you have pain, you know you have to kind of deal with it.

PA-MI

## Discussion

### Principal Findings

Overall, this qualitative study provides strong insights from patients with genetic disorders into the challenges of receiving quality care from health care providers, managing their health condition, and finding pain mitigation techniques.

To the best of our knowledge, this study is among the first to explore the challenges faced by patients with genetic disorders with health care systems and their efforts to find solutions and treatments from the internet via online support groups. The results demonstrate that most patients with genetic disorders are disappointed with the help they receive from their health care providers and actively looking for solutions through different academic and web-based resources. Among all resources, most participants agree that online peer support groups have changed the way they were managing their health previously and helped with care management improvement. The findings from this study can be used to inform future studies on the development of new or improved online support groups. In addition, the findings can be used to reduce the gap of information between patients and health care providers.

### Comparison With Previous Studies

This study distinguished the utility of traditional physician-patient relationships versus online support groups in obtaining helpful information for the management of EDS symptoms. The focus of this study was to explore the relationship between people and the medical community when the medical community has limited knowledge of a person’s disease. We focused on EDS as a representative of the large community of people with genetic disorders and other rare diseases.

All participants (30/30, 100%) reported experiencing consistent pain, which had major impact on their daily life. Interacting with members of the medical community—general physicians, specialists, chiropractors, and physical therapists—left the study participants dissatisfied with the information they were receiving. From inaccurate diagnoses to lack of knowledge about EDS to recommending treatments that would only worsen the symptoms, trust in health care providers was generally lacking. Owing to frustration, these participants turned to alternative sources of information that they were able to find in online support groups. They found these groups to be helpful places for finding information to manage their conditions. Our findings from this study appear to be consistent with those of previous studies regarding the positive effects of support groups in pain education and the increase in the patient’s knowledge about their disease [21-24].

Primarily, study participants were looking for information in 4 categories. First, they wanted to know more about their disease and were interested in any new study that was available in medical journals and publications. Second, they were interested in referrals for effective care providers and products such as braces or topical creams. Third, participants were seeking advice for dealing with everyday challenges such as driving or sleeping comfortably. Finally, participants were looking for emotional support from other people with EDS. Previous studies have demonstrated that patients use support groups to write about their pain and express their feelings. Researchers have found that patients were learning about pain management techniques and reducing their dependence on pharmaceutical pain management tools such as opioids [25-27].

All study participants (30/30, 100%) were very actively engaged in managing their health, and most of them (28/30, 93%) had found that the way they managed their health had been positively affected by participating in online support groups. Participants cited emotional support in using a mobility device, discovery of new bracing techniques, learning self-advocacy in relation to care providers, and generally improved self-reliance as some of the ways online support groups helped them to manage their health.

Study participants mainly resided in the United States, but were also residents of Italy, Canada, Sweden, the United Kingdom, Australia, and the Netherlands. Participants in the United States were slightly more likely to have a positive experience within the health care system, but attitudes toward caregivers and online support groups were similar and independent of country.

Although overall opinions of online support groups were positive, most study participants (24/30, 80%) had significant reservations about them. A common experience was the ineffective ways in which information was presented. Many participants (23/30, 77%) expressed dismay at the amount of information they would have to screen to find the specific information they were looking for. Most participants (25/30, 83%) expressed concern about the veracity of the information they were finding and the lack of citation of a trustworthy source. Many participants (20/30, 67%) found the tone of the support groups to be negative, either owing to self-pity or unwarranted criticism of other group members. Most participants (15/30, 50%) also expressed concern about security and privacy and often preferred to be anonymous. Our findings from this study are compatible with those of previous studies regarding patients’ lack of privacy and the need for better information dissemination formats [28,29]. Several other studies have focused on the design and technical aspects of online support groups. They stated that web-based platforms should be more user-friendly and help patients to navigate and access information easily [30,31].

Most study participants indicated that health care professionals did not assist them with or direct them toward the online support groups they ended up using. Health care providers were not aware of the benefits of the information available on these sites. Providers were also not aware of the vast amounts of clinical information that users were compiling on online support groups that would be useful in studying EDS. Given that the economic burden of 379 rare diseases in the United States in 2019 was estimated to be approximately US $1 trillion, health care providers can help to reduce these high medical and nonmedical expenditures by referring patients to online support groups [32]. On the basis of the Centers for Disease Prevention and Control, the total costs of top three chronic diseases such as diabetes [33] (US $327 billion in 2017), cancer [34] (US $157 billion in 2020), and heart disease and stroke [35] (US $214 billion in 2018) are far less than the costs of rare diseases.

Future investigations into online support groups will focus on the ways these sites can be structured to directly meet the needs of patients who are chronically ill.

### Limitations

A key limitation of the study was the small sample size and self-selection of participants through one website (The Ehlers-Danlos Syndrome Society). Most participants were women (28/30, 93%), and most were White (28/30, 93%). All the participants (30/30, 100%) were English speakers. In addition, we focused only on one rare disease. A study that includes a broad sample of rare diseases using multiple survey instruments across time can provide valuable insights regarding the role of online support groups in the management of rare diseases. Our findings demonstrated the potential value of online support groups in helping patients in managing their health conditions. Further studies may focus on the existing features of current support groups’ platforms, *missing* features, and how developers, with the help of health care providers, can improve their platforms to reduce the spread of misinformation and increase the credibility of contents.

### Conclusions

Professionals providing care to patients with EDS and other patients with long-term debilitating conditions should consider online support groups as a source of information for assisting their patients with their conditions. By participating in the same sites as their patients, professionals will be able to provide context to the information being posted, and they will add some trustworthiness to the content. These professionals will also gain insight into the needs of their patients that are not being met in the clinical environment.

## Appendix

Multimedia Appendix 1 Interview question guide.

## Abbreviations

EDS: Ehlers-Danlos syndrome
