# Supplementary material for: The Internet Knows More Than My Physician: Qualitative Interview Study of People With Rare Diseases and How They Use Online Support Groups
Source: J Med Internet Res. 2022 Aug 25;24(8):e39172. doi: 10.2196/39172 (PMC9459833; doi:10.2196/39172)
Supplement: Multimedia Appendix 1 [file jmir_v24i8e39172_app1.docx]

Patient Experience-Based Information Systems Application for Mitigation of Genetic-Disorder Pain

INTERIVEW

My name is Sadaf Ashtari, and I am an Assistant Professor at California State University, Sacramento, College of Business, Information Systems Department. I am conducting this research study to measure patient experiences with, and feelings about, pain management through social media.

If you volunteer, you will be asked to answer some audio-recorded interview questions that will take about 20-30 minutes.

If you agree to participate, you can stop at any time. Risks of participation are not anticipated to be any greater than risks you encounter in daily life. There may be some benefits to this research, particularly that results may provide a better understanding of social media/support groups among genetic-disorders patients and how that affects pain management.

I intend to publish or present my results. You will not be identified in my results. I will protect your identity by (1) grouping responses/using pseudonyms, (2) storing collected information in a protected location, and (3) removing identifiers as early as possible. Information that can identify you will be deleted or removed from the data after a period of 3 months. The de-identified data will be kept in a secure location and may be used for other research studies. I will destroy the de-identified data 3 years after the study ends.

If you have any questions about the research at any time, please contact me at [sadaf.ashtari@csus.edu](mailto:sadaf.ashtari@csus.edu) or by phone at 916-278-7102. If you have any questions about your rights as a participant in a research project please call the Office of Research, Innovation, and Economic Development, California State University, Sacramento, (916) 278-5674, or email irb@csus.edu.

Your verbal consent implies that you have read and understand this information and that you may stop at any time without penatly.

Please keep this form as your copy.

**Interview Questions:**

Demographic:

- What is your gender?
- What is your age group?
- What is your racial or ethnic identification?
- What’s the highest degree or level of education you have completed?
- Are you currently employed? If yes how many hours per week do you Usually work?
- Where do you live? US or other country-

1. How long have you known about your disease?
   1. How often you are in pain?
2. What are your approaches for pain management?
3. Where do you usually find help about your condition?
   1. If you want to learn more about your disease/condition/pain management, what do you do?
   2. What sources have you found useful/useless in providing help about managing your condition?
   3. What technology(ies) or application(s) do you usually use?
   4. Are you part of any social media support group(s)?
      1. How do you feel about it/them?
      2. Do they help?
   5. What types of information are you looking for on these apps/websites?
4. Did anyone in the healthcare system tell you about any website/applications/support groups?
5. Does pain impact your usage of technology/social media? In what capacity?
6. Has the way you manage your health changed as a result of participating in the online support group? If so, how?
